# Abdominal Movements with Reference to Pregnancy—their Nature and Cause

**Published:** 1848

**Authors:** J. E. McGirr

**Affiliations:** Professor of Anatomy, Physiology, and Chemistry, in the University of the Lake, Chicago


### ARTICLE III.

*Abdominal Movements with reference to Pregnancy—their Nature and Cause.* By J. E. McGirr, A.M., M.D., Professor of Anatomy, Physiology, and Chemistry, in the University of the Lake, Chicago.

Since the article on this subject, that appeared in the last No. of the Journal, was written, I have seen the detail of a case given by Prof. G. S. Bedford of New York, in an introductory lecture, which bears painfully yet strongly in favor of my proposition, *that it is an error to regard abdominal movements as certain evidence of pregnancy.*

A young lady, the daughter of a venerable clergyman of the Church of England became engaged to a young "barrister of promise and respectability. Soon after the engagement she began unaccountably to decline in health. There was considerable irregularity in her monthly periods; with nausea, loss of appetite, inability to sleep, feverishness, an uncontrolable dislike to society. There was also a marked change in her personal appearance, her abdomen became enlarged, her breasts increased in size, &c., &c." Her female acquaintances, noticing these changes, gave the report to the winds that they were the result of pregnancy. The young barrister to whom she was affianced, hearing them begged to be released from his engagement. This was done at once. The young lady, conscious of her innocence, was willing to submit herself to examination by a physician. One was called, and when he found "movements" added to the symptoms above detailed, he pronounced her pregnant. The father, indignant at these imputations upon his daughter's character, called in a second. His opinion was that she was pregnant. The poor, distracted old man, gathering up what of the world's goods he had, determined to start for America, where he would be beyond the reach of scandal; for he never doubted his child's innocence.

On the voyage out, she became "extremely ill," the physician on board being called to her, pronounced that "there was danger of premature delivery." When they reached New York the case was submitted to Dr. Bedford, who, after an examination made *scientifically*, pronounced, that "she was *not* pregnant.

But the balm thus poured into the father's heart could not save his beloved child from the wreck which the hasty opinion of her English physicians had wrought for her. Shame, and disappointment, and anxiety had made fearful ravages upon her delicate constitution, and she was now an incurable victim of consumption.

About four weeks from the date of this examination the Dr. was called to make a *post mortem* examination upon her. "The father stood by and witnessed every stage of the operation; his form was erect, his face pale and thoughtful, and one tear would have broken the agony of his grief. As the tumour was removed from the womb he seized it convulsively and exclaimed. 'This is my trophy; I will return with it to England, and will confound the traducers of my child.'"

But this act, which might calm his paternal grief, could not move the now still heart or mantle a blush upon the cold cheek of his immolated child. Yet what remorse must not the sight of that "fibrous tumour" swell up in the hearts of those men over the ocean, when it would be evidence to them that their erroneous decisions had sent a lovely victim to an untimely grave. If they had the feelings of men they would rather have been in the lowest depths of the sea than to have seen this damning evidence of their blunders.

A medical friend has related to me a case which also seriously illustrates the consequences which may result to the accoucheur who considers the presence of these movements as direct evidence of pregnancy.

A lady supposing herself to be in her sixth month of pregnancy, became unwell. She sent for her physician, who, since she had some manifestations of approaching labor as she supposed, had treated her accordingly. The uterus was distended and the motions of the child distinct. She had no doubt of her pregnancy, and her physician had none. She was attacked with a severe flooding, which returning three times during a week, reduced her so low that she died before the birth was accomplished. Her husband allowed a post mortem examination to be made; but what was the astonishment when the uterus was opened to find it filled with hydatids. There was no child there!!

I have yet a few observations to offer upon the nature and causes of these movements, premising that I am indebted to Dr. T. Smith, the eloquent lecturer on the physiology of parturition, for many of my facts.

Females describe these movements as of two distinct kinds. First: they will tell you that in directing the hand over the abdominal walls, they will feel irregularities like projecting portions of the child: that these give rise to wave-like movements at the several points simultaneously and often occasion pain; and that sometimes upon merely touching one of these prominences, the child will kick so violently and so long as to render the abdomen sore and almost to rend its walls. Secondly: they will tell you that they have felt a sensation like that of an infant starting in its sleep, or like the sensation produced by a moderate electrical shock.

The first and most frequent of these motions is uterine. The second is that of the child. These movements commence at the period of quickening, and manifest themselves sensibly to the accoucheur at the fifth or sixth month.

At the latter period of pregnancy these movements are of so great violence that the term kick has been applied to them. At one time the kick is experienced at one part of the abdomen, then at another and another, until we would think the child all heels or that there was a litter of children. Sometimes the strokes are so violent as to move the bed clothes over the abdomen, or to cause the hand to rebound. Now if the child turned about in this way the chord or the placenta could not escape injury, nor could the presentations be so generally favorable—wrong presentations would be the rule.

A moment's reflection will show us that all these movements cannot be referred to the child. It, as I have already observed, may be dead, or no child be present; the uterus may be distended by hydatids, and yet, motions may be present so as to deceive the female and her physician, or no motions may be perceptible, and still, a living child may be born.

Again, it seems an absurdity to endow a child, at the age when these movements begin, with strength sufficient to inflict so much annoyance. Indeed we sometimes find the puniest, skeleton-like little wretch charged with being the cause of more than usual disturbance; while a large and healthy child will be supposed to be less restive. If these movements depended upon the child, the reverse should happen.

Dr. Smith says there is no motor power which could excite these almost perpetual motions in the fœtus. Volition, cerebral voluntary motion, before respiration has been set up, and the brain roused by arterial blood, could give rise to none, nor could emotion. Reflex actions, from the nature of the child's position in the liquor amnii, would be faint indeed.

The instances recorded of "anencephalic and amyelitic" fœtuses show that these supposed movements may occur equally well when there is neither brain, nor spinal marrow present. In these cases the two "great sources" of reflex and voluntary motor actions never existed. Now if the movements were fœtal and the result of motor action—we would be obliged to refer that motor action to the ganglionic nerves. That would be nonsense.

To the uterus then, and not to the child in the uterus must be assigned the seat of these movements. The instances already given shows that it cannot be the child that causes them. Now we know from observation, that the uterus is affected by a wave-like, peristaltic motion, which is marked, and which may be easily seen: and it certainly is not improper to allow the uterus, a muscle itself, the privilege of occasionally manifesting the property of contraction. With our present ideas we are forced to believe that it grows passively. It does not follow that its condition requires it to remain at rest; for the contraction may be partial, and the peristaltic motions are very different from those contractions that effect the expulsion of the child.

If, then, these motions do not depend upon the child, if they are to be referred to a peristaltic action of the uterus itself, the importance of being certain whether there exists at all, a child in utero, or, if there does, whether the child be alive when the alternative is that of an operation, becomes at once evident: and the accoucheur, who values his reputation, his peace of mind, or the life of a fellow being, will never neglect so important a means of reducing doubt to certainty, as that which modern science has placed in his hands.

